# Coping strategies, challenges and potential interventions among adult patients with HIV and mental illness comorbidity in southwestern Uganda

**DOI:** 10.1080/17441692.2024.2372802

**Published:** 2024-07-11

**Authors:** Prosper Katugume, John Bosco Namukowa, Oliver Nankunda, Trevor James Muhwezi, Ruth Namaseruka, Edith K. Wakida, Celestino Obua, Nathan Kakongi

**Affiliations:** a Faculty of Medicine, Mbarara University of Science and Technology, Mbarara, Uganda;; b Department of Nursing, Faculty of Medicine, Mbarara University of Science and Technology, Mbarara, Uganda;; c Department of Medical Laboratory Science, Faculty of Medicine, Mbarara University of Science and Technology, Mbarara, Uganda;; d Department of Pharmaceutical Science, Faculty of Medicine, Mbarara University of Science and Technology, Mbarara, Uganda;; e Office of Research Administration, Faculty of Medicine, Mbarara University of Science and Technology, Mbarara, Uganda;; f Department of Medical Education, California University of Science and Medicine, Colton, CA, USA;; g Department of Pharmacology and Vice Chancellor, Mbarara University of Science and Technology, Mbarara, Uganda;; h Department of Biochemistry, Faculty of Medicine, Mbarara University of Science and Technology, Mbarara, Uganda

**Keywords:** Coping strategies, challenges, potential interventions, HIV-mental illness, comorbidity, SDG 10: Reduced inequalities, SDG 15: Life on land, SDG 1: No poverty, SDG 3: Good health and well-being, SDG 5: Gender equality

## Abstract

HIV and mental illness comorbidity presents significant healthcare challenges, especially in low- and middle-income countries where healthcare systems often address individual conditions rather than comorbidities. This results in poor coping, increased vulnerability and diminished health-related quality of life. This study investigated coping strategies, challenges and potential interventions for individuals with HIV-mental illness comorbidity in Southwestern Uganda. The study included purposively selected people with HIV and mental illnesses seeking care in health facilities across Southwestern Uganda. Data from in-depth, semi-structured interviews were transcribed verbatim and entered into ATLAS.ti-7 for analysis. Thematic analysis was employed, generating codes from the transcripts to develop themes. The data revealed three categories: coping strategies, challenges and potential interventions. Three key coping strategies emerged: conscious avoidance of emotional stressors, maintaining emotional stability through social interactions and reliance on prayer. Challenges included social isolation, financial crises, vulnerability to abuse and medication management issues. Respondents recommended scaling up mass educational programmes to increase awareness of causes, preventive measures and association between the two comorbidities, together with implementing financial aid initiatives as viable interventions. These findings highlight the importance of addressing comorbidities together for improved emotional stability and underscore the value of the proposed potential interventions for healthcare systems and policymakers.

## Introduction

The comorbidity of HIV and mental illness has been documented since the early 1980s when HIV was first diagnosed (Atkinson et al., 1988; Perry & Tross, 1984). Subsequent studies have consistently reported a higher prevalence of mental illness among HIV-infected populations compared to the general public (Hoare et al., 2021). However, the extent of this comorbidity varies from one country to another and in terms of the type of mental illnesses involved. The global prevalence of HIV was 39 million people by 2022 (UNAIDS, 2022), of which 65% reside in Sub-Saharan Africa (van Schalkwyk et al., 2024). In Uganda, the current prevalence of HIV stands at 1.4 million people, with the adult (15–49 years) prevalence of 1,350,000 (5.4%) and new infections of 52,000 annually (Uganda-AIDS-Commission, 2023). With mental illness, the global prevalence was 970·1 million cases by 2019, with depression and anxiety being the highest globally at 3440 and 3780 cases per 100,000 people, respectively, and 4540 and 3463 in sub-Saharan Africa (Mental_Disorders_Collaborators, 2022). In Uganda, the prevalence of mental illness in adults is 24.2% (95% C.I 19.8% – 28.6%) according to Systematic Review and Meta-Analysis by Opio et al. (2022). The prevalence of HIV-mental illness comorbidity is not well documented, but a systematic review of 142 studies conducted in the USA, sub-Saharan Africa, Europe and Asia revealed a high prevalence, with studies reporting 28−62% of PWH having mental health symptoms (Hoare et al., 2021). In sub-Saharan Africa, which bears the highest burden of HIV globally, the prevalence of mental disorders among PWH is equally high, ranging from 14.2% to 28.1% (Kagee et al., 2017; Motumma et al., 2019; Ngum et al., 2017). In Uganda, the prevalence of HIV-mental illness comorbidity is not well documented although Mpango et al. (2023) revealed 8.2% HIV prevalence among patients with severe mental illness. Some studies have shown higher prevalences of specific mental disorders in HIV. A systematic review and meta-analysis of 43 studies (Kaggwa et al., 2022) revealed 28.2% of PWH having depression, while the Chaka study involving 1339 randomly sampled children and adolescents with HIV in the central and southwestern regions of Uganda revealed 13.1% for ‘any anxiety and depressive disorders’ (Mpango et al., 2022), ‘any DSM-5 psychiatric disorder’ of 17.4% (Kinyanda et al., 2019) and suicidality of 10.7%.

For the purposes of this study, mental illness refers to any of the commonly experienced mental disorders including depression, anxiety and posttraumatic stress disorder, schizophrenia, bipolar disorder and other psychotic conditions (Kakhramonovich, 2022; Koschorke et al., 2021).

Individuals with comorbid mental illness and HIV typically experience multiple stressors related to their conditions. These stressors encompass poor adherence to medication, HIV-related stigma, loneliness, reduced quality of life, accelerated HIV disease progression and heightened risky sexual behaviours, both in terms of acquiring and transmitting the infection (Abas et al., 2018; Areri, 2019). These challenges could potentially impede patient engagement in various healthcare delivery strategies, resulting in poorer health outcomes, such as reduced viral suppression and stressful life proceedings (Vitale & Ryde, 2018). Equally, the stigma towards mental illnesses usually acts as a barrier to accessing HIV treatment, which impacts treatment outcomes (O’Grady et al., 2024) that include morbidity and mortality from HIV comorbidities (Taiwo et al., 2023).

In Uganda, the integration of HIV and mental illness comorbidities into the healthcare system still faces challenges. HIV and mental illness are often managed independently in the country, placing the responsibility of coping with HIV and mental illness comorbidity primarily on individual patients. This is likely to expose them to adverse outcomes. Additionally, there is a lack of readily available evidence regarding coping strategies for individuals with HIV and mental health comorbidities in Uganda. Therefore, this study is aimed to explore, using qualitative methods, the strategies employed to cope with HIV and mental health comorbidities within communities in Southwestern Uganda. The study also identified the challenges these individuals usually encounter and suggests potential interventions that can enhance their quality of life.

## Methods

### Research design

This was a descriptive phenomenological study conducted using individual, in-depth, semi-structured interviews to explore coping strategies, challenges and potential interventions among adult patients living with comorbid HIV and mental illness.

### Study setting

The study was conducted at two health facilities: Kabuyanda Health Center IV (HCIV) in Isingiro district and Kitagata General Hospital in Sheema district. The two health facilities are approximately 319 and 347 km, respectively, from the capital city, Kampala; 49 and 77 km from Mbarara city, the major city located in the centre of southwestern Uganda. The Health Center IV offers a wide range of healthcare services, including preventive, promotive, outpatient curative, maternity, inpatient care, emergency surgery, blood transfusion and laboratory services, serving a catchment population of 100,000 people (Uganda-MoH, 2018). The general hospital has more services than the Health Center IV but both offer HIV and mental health services, especially on clinic days designated for either condition despite the two being handled independently.

### Participants

Adults aged 18 years and above, living with comorbid HIV and mental illness, attending healthcare services at the two selected health facilities, participated in the study. A total of 21 respondents, ranging from 22 to 62 years, were included in the study. This group comprised 9 males and 12 females. All participants had previously received comorbid diagnoses for both HIV and mental illness, and their mental capacity to participate in the study was assessed twice by a psychiatric nurse, and then by the research assistant. Mental capacity, as defined by Lundberg et al. (2013), includes the ability to understand information about the study, the capacity to express a choice based on given information, insight about having a mental health problem and the ability to participate in an in-depth, semi-structured interview. Patients who were giving uncoordinated speech due to mental relapses were excluded from the study.

### Sampling criteria and selection

With the assistance of a psychiatric nurse and the antiretroviral therapy (ART) clinic in-charge nurse at the health facilities, potential respondents were identified through their records as they attended ART and psychiatric clinic reviews. Criterion sampling was used to enrol adult patients with both HIV and mental illness records, who were accessing healthcare services and capable of providing the required information. To ensure heterogeneity in the sample and capture diverse ideas, a comorbid diagnosis of one month and above was considered. In order to recruit stable patients with no active symptoms, mental capacity was first examined by the psychiatric nurse using the DSM-5 Mental status examination.

### Data collection procedure

Data were collected through individual, in-depth, semi-structured interviews using an interview guide that researchers generated specifically for this study, aligning with the study’s objectives. This guide was pre-tested on four respondents living with HIV-mental illness comorbidity at Mbarara Regional Referral Hospital, which is outside the study area. Following the pretest, minor adjustments were made by the investigators to enhance the clarity of interview questions and capture the necessary information. Two research assistants, who conducted the interviews, had backgrounds in nursing and public health and experience in qualitative data collection, and were first trained by two lead investigators. To ensure privacy, data were collected in a private room secured at each facility. The interviews were audio-recorded and supplemented with field notes. Each interview lasted for an average duration of 40−50 minutes. Data were collected until saturation of data was achieved (Creswell & Poth, 2016), and this was observed with the last three interviews. The interviews were conducted in either the local language (Runyankore) or English depending on the convenience of the respondent. The interview guide had open-ended questions majorly focusing on knowledge, period with the two conditions, coping strategies used to live well with the comorbidity, enablers of coping, challenges encountered in coping and potential interventions that can enhance coping.

### Data management and analysis

The recorded data were transcribed verbatim and translated from the local language into English by the research assistants. To avoid personal judgement and belief during transcription and translation, the data collected (audio and field notes) were exchanged between RAs to ensure some blindness. After the translation, all the data were obtained including deletion from the RA gadgets by the author (NK) for safe custody and confidentiality. The lead researchers, represented by the two main authors (PK and NK), initially read all the transcripts to familiarise themselves with the data (Muhr, 2013). Inductive thematic analysis was employed, generating codes from the transcripts to develop themes. The two main authors (PK and NK) used eight samples of transcripts to develop the codes based on responses in the transcripts. To ensure intercoder reliability, PK and NK independently developed codes and code definitions before harmonising them. All the data (transcripts) were imported into ATLAS.ti, version 7, a qualitative data management software, and analysed based on the codes and code definitions. Data segments were then retrieved, sorted, described and interpreted based on recurring patterns and themes that emerged from the analysis and the study’s objectives. Unclear data segments were labelled and revisited several times looking for similarities with other segments. Very unclear data segments were left out in the analysis, but those with similarities were categorised in relevant themes.

## Results

### Socio-demographic characteristic of study respondents

A total of 21 respondents, ranging in age from 22 to 62 years, were included in the study. This group comprised 9 males and 12 females. Among the respondents, five had no formal education, eight had primary education, six had completed secondary education and two had attained a tertiary education level. Regarding marital status, nine respondents were married, four were widowed, seven were divorced and one was single.

**Figure 1.**
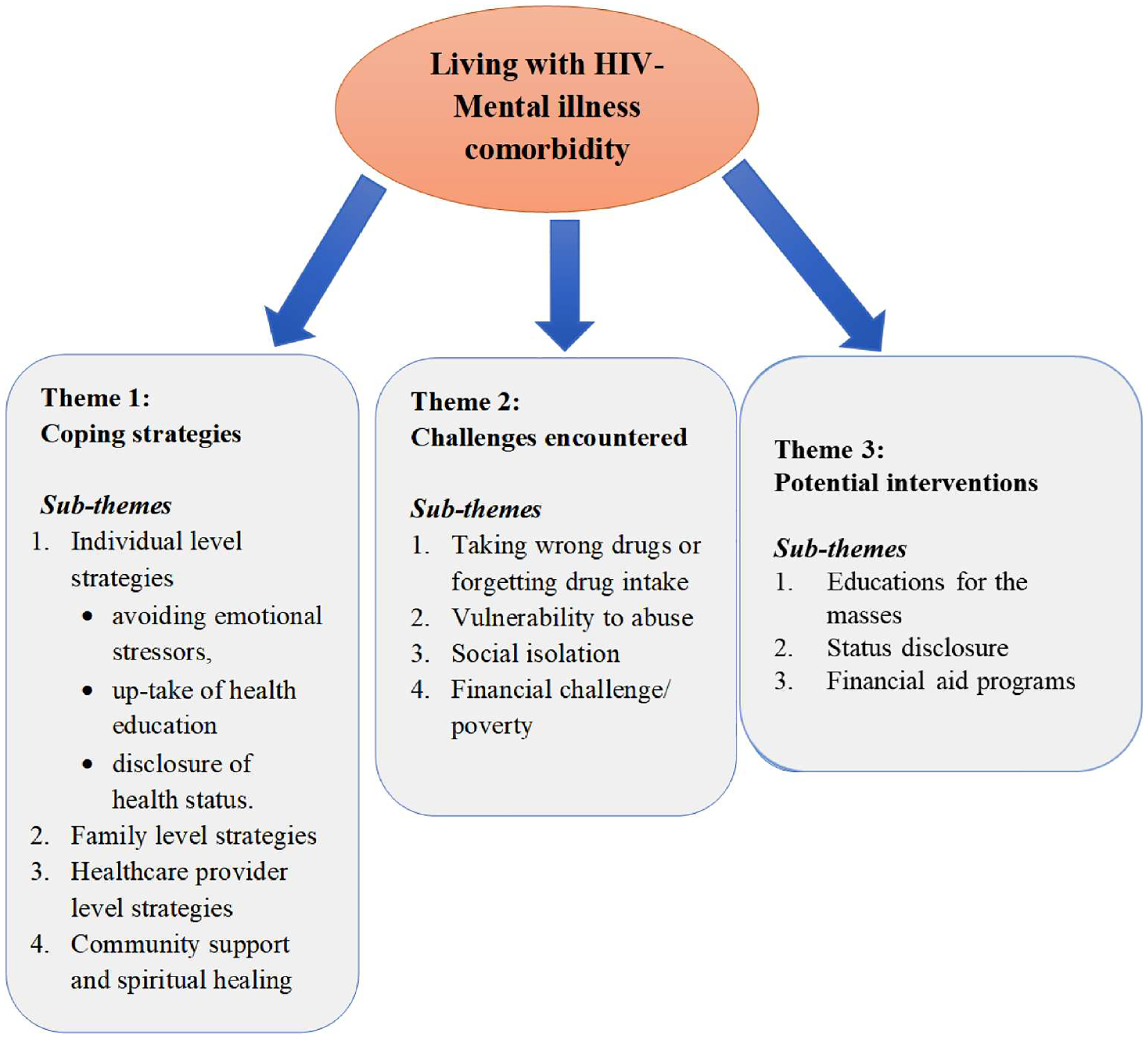
Coping strategies, challenges encountered and potential interventions for People with HIV and mental illness comorbidity.

Our findings have been organised into three overarching categories (1) coping strategies, (2) challenges encountered and (3) potential interventions. These results displayed significant variability and were further subdivided into sub-themes that emerged during the analysis, as summarised in Figure 1.

### Coping strategies

The coping strategies reported have been categorised into four sub-themes that emerged from the analyses: individual, family, healthcare and community-level strategies.

#### Individual-level strategies

At the individual level, the respondents reported employing three primary coping strategies: avoidance of emotional stressors, uptake of health education and disclosure of health status.

##### Avoidance of emotional stressors.

The study revealed that finding peace of mind by avoiding emotional stressors such as quarrels, worries and annoyance, as well as valuing quiet time and having self-belief, helped many patients effectively cope with the comorbidity of HIV and mental illness.

… I don’t want anybody to talk to me badly, I don’t want anybody to disturb me, if anybody sees me in wrong, they better let me fall in that bad trap or use soft ways to rescue me from the wrong but not to force me.

(Male, 42 years)

##### Uptake of health education talks.

Many of the respondents acknowledged that health education talks have enhanced their comprehension of how other conditions, including mental illness, tend to coexist with HIV. They emphasised the importance of attending these talks. Some knew that mental illness is a result of HIV medications, while others believed it was linked to having many thoughts. The verbatim quote below illustrates this.

I was started on both ART and mental illness drugs, teaching me all the directions to follow and I really follow them well. They usually teach us that sometimes mental illness is a result of HIV, so they advise us to always take HIV drugs in time.

(Divorced female, 38 years)

##### Disclosure of health status.

Most of the respondents indicated that being open and disclosing their HIV status and mental illnesses gives them peace of mind and allows them to live harmoniously with others. However, a few reported that even when they do not disclose their mental illness, people tend to notice it on their own.

… you know, if you want to live peacefully with these things (meaning HIV and mental illness), let people know. … for me I tell people my problem (meaning HIV and mental illness), because if I hide them and they kill me they will say that it is malaria. …. so to keep myself safe, I tell them so that when I am down, they know where to take me but not to die thinking it is malaria.

(Male, 38 years)

#### Family-level strategies

The majority of the respondents reported that when their family members and relatives understand both health problems, it becomes easier for them to manage the burden of HIV and mental illness comorbidity, as they can access the necessary support. However, some respondents mentioned instances of abuse or harassment by their family members.

….. I have a wife who took the initiative to understand my two conditions and she understands together with my children how my attack of mental problem begins and so they do what they can to help me.

(Male, 42 years)

#### Healthcare provider-level strategies

The majority of respondents emphasised the significance of the healthcare system, particularly the provision of medications for both conditions along with counselling. These services have greatly assisted them in coping with the comorbidities. They are particularly motivated by the fact that healthcare providers sometimes visit them at home.

…. my counselor is always there for me, and if anything disturbs me, it is where I rush to. And at times they follow us to our homes to ask why we are not picking our drugs and they give us.

(Male, 36 years)

#### Community support and spiritual healing

The majority of the respondents praised social interactions, such as participation in church activities, community social events and interactions with individual community members, for significantly contributing to their overall well-being. When asked about the church’s role in helping them cope with the two comorbidities, most of the respondents acknowledged the significance of prayer and educational healthcare discussions in maintaining their emotional stability, as evidenced by the following quote.

… they teach us a lot when they call us for trainings at the church, like telling us to love ourselves, engaging ourselves in money generating activities, and others. … And when they pray for you, you feel healed.

(Female, 45 years)

### Challenges encountered in coping

Patients dealing with the comorbidity of HIV and mental illnesses face a range of challenges as they strive to maintain their well-being while managing these conditions. Four sub-themes have emerged, categorising the challenges under each of these themes.

#### Taking wrong medication or forgetting drug intake

Mental illness re-occurrence, bad dreams and hallucinations were reported, and are attributed to the mental illness itself, which causes them to take the wrong drugs or forget altogether as shown by the verbatim quotes below:

Individuals have reported experiencing mental illness re-occurrence, bad dreams and hallucinations. These issues are often attributed to the nature of mental illness, which sometimes leads them to take the wrong medications or forget to take them altogether. This is evident in the following verbatim quote:

…. when my head is upset and tells me to go, I go and leave my drugs behind. When I reach like in Mubende (a district 300 km away) and get settled mentally, I remember the drugs. … … there is a time I moved away with a wrong drug and got a skin rash after taking it.

(Male, 36 years)

#### Vulnerability to abuse

Some individuals have exploited the vulnerabilities of comorbid patients, including self-proclaimed spiritual healers, often referred to as ‘prayer warriors’. These individuals have taken advantage of patients with mental illnesses and, in some distressing cases, subjected them to sexual abuse, which, for certain patients, was the root cause of their HIV infection. This disturbing revelation was made by a respondent who contracted HIV from a prayer warrior who had initially committed to providing care and offering prayers at his home.

… I got it (meaning HIV) from a pastor (prayer warrior) who requested my parents and took me to his home to keep praying for me, but he died (Respondent cries).

(Female, 22 years)

#### Social isolation

Social isolation emerged as a prevalent challenge. Some of the respondents find themselves in isolation, while others have lost their close friends, as indicated in the following quote:

… yes, I meet challenges. After they (meaning friends) have discovered that you are sick, they run away from you. For example, where I stay, I am isolated and I care for myself because I have discovered that my neighbors are now my enemies, me I am alone with my God.

(Female, 48 years)

#### Financial challenges and poverty

The majority of respondents cited poverty as their primary issue, compelling them to engage in excessive work or strenuous activities, such as full-day labour in others’ gardens, for the sake of survival. This economic strain often exacerbates their health conditions.

… I always get worried, because I don’t have my own house and I don’t have money to buy even a mattress. I also work too much like digging for others in order to get some money to survive on, and this has led to my poor health.

(Female, 38 years)

### Potential interventions

To improve the quality of life for individuals living with HIV and mental illness comorbidity, respondents were asked for their opinions on what could be done to enhance coping and mitigate the challenges they face as they live with these comorbidities. Three sub-themes emerged, and the findings are presented under these sub-themes.

#### Education for the masses

While respondents indicated a lack of awareness regarding the relationship between HIV and mental illness comorbidities, they called for government and religious institutions to initiate educational efforts aimed at informing the general public about the causes, risks, relationships and preventive measures for these comorbidities. These observations are supported by the quote below:

Government and the church should provide education to all people on the causes of these diseases and how to prevent them. … … Maybe people can be careful not to get them or spread them.

(Female, 45 years)

#### Status disclosure

To enhance coping with HIV and mental illness comorbidity and to avoid related challenges, the majority of the respondents reaffirmed that disclosing their status to others makes them feel more liberated and enables them to access the necessary assistance. For instance, in response to the question: In your opinion, what can be done to help people living with both HIV and mental illness cope well and lead fulfilling lives? One respondent indicated that:

… people should always disclose their status to community members to be helped, and people in community should also help their fellow sick members. …. There you live a very free life.

(Female, 50 years)

#### Financial aid programmes

Providing financial aid to those who are ill, establishing income-generating projects for them, or facilitating their employment has been suggested as a potential intervention to address financial challenges and enhance coping. These observations are supported by the quotes below:

… the rich people or government should help the sick by giving them some money to keep them surviving just as they do for the elderly. I wish they could give every sick person at least some money to help them live a better life.

(Male, 44 years)

One respondent summarised their thoughts on what can be done to improve coping with HIV and mental illness comorbidities. Their narrative suggests that abstaining from sex, maintaining good nutrition, adhering to treatment, as well as seeking support through prayers and counselling can significantly contribute to a better life for individuals dealing with this comorbidity.

… stop moving around with men/women despite being infected, they should eat and drink enough. Also, let all people test themselves because once you find that you are sick, you get medical advice and take drugs very well, and you can always move with your drugs so that you take them on time and you don’t miss. …. those who are mentally ill should always go to hospital for drugs and should take a lot of water. Church should always come to pray for those people and counsel them.

(Male, 50 years)

## Discussion

This study was conducted to develop a clearer understanding of the coping strategies employed by people living with comorbid HIV-mental illness, the challenges they encounter during coping and potential interventions that can enhance their quality of life. The phenomenological evidence generated is intended to provide a basis for devising better healthcare interventions that can minimise challenges associated with HIV and mental illness comorbidities and increase competence in coping strategies for better healthcare outcomes among adult patients.

The observed coping strategies encompassed a range of approaches, including intuitive actions at the individual level, empathic actions within the family or community and professional interventions at the healthcare facility level. All of these strategies worked together to help patients cope with the challenges of having both HIV and mental illness. The challenges encountered in attempting to cope with this comorbidity included social, financial, vulnerability and forgetfulness issues. However, respondents also provided suggestions for potential interventions that could mitigate these challenges and improve the overall quality of life.

We discovered that some respondents intuitively employed coping strategies by avoiding emotional stressors, such as quarrels, worries, and annoyances and by creating time for quiet moments. This avoidance of emotional stressors, as described by the respondents in our study, represented an instinctive means of preserving their psychological and emotional well-being, commonly used when encountering uncomfortable situations as reported by Huda et al. (2021) highlighting emotional-focused coping to mediate the relationship of stress with physical and emotional well-being. The positive emotions helped them build personal coping abilities when dealing with the challenging circumstances of HIV and mental illness comorbidity. According to the respondents, these strategies provided them with peace of mind, a crucial factor in enhancing the quality of life and overall health for individuals dealing with mental illness and HIV. This perspective aligns with findings from the study by Ramirez-Garcia et al. (2019) and Rentala et al. (2015) who noted that mind–body practices are frequently used by people living with HIV to reduce symptoms and improve well-being.

Disclosing one’s status was reported as a strategy that helped many to cope with the psychological and emotional effects of the two illnesses as it gives them peace of mind and allows them to live harmoniously with others. Similarly, other studies reported that disclosure of HIV status leads to positive outcomes like reducing gossip and rumours, support from loved ones and community members (Makoae et al., 2008) although certain factors for disclosure vary across types of disclosure targets (Ma et al., 2023).

Empathy is essential for human social behaviour, where evidence shows that enhanced empathy supports coping with psychosocial stress (von Dawans et al., 2022). In line with this, our findings also show that an understanding and empathetic family or community supported the respondents to cope with the challenges of HIV and mental illness comorbidity. Utilisation of family support may be at individual level from a relative or family as a whole in providing care ranging from assisting the individual to get right mental and HIV health services, identifying presentation of mental illness symptoms, assistance with daily living, reminders on drug intake and financial assistance continuous information provision (Areri, 2019; Buckingham et al., 2013; Buh et al., 2023; Nguyen et al., 2019; Wouters et al., 2016). Moreover, increased reliance on other people for social support has been reported as a key tool for improving ART adherence and daily functioning (Buh et al., 2023; Reynolds et al., 2022).

We found that individuals with HIV and mental illness comorbidity commonly used coping strategies like prayers and social interactions. These strategies have been previously reported to influence healthcare-seeking behaviours, particularly in rural areas (Kakongi et al., 2020). Spiritual support and social interactions also play a vital role in providing emotional stability, reducing stigmatisation and helping individuals cope with the challenges associated with HIV-mental illness comorbidity (Areri, 2019).

Participants frequently reported attending counselling sessions at healthcare facilities as a coping strategy. This approach provides psychological support, aligning with previous research (Hoare et al., 2021). Self-management through emotional and psychosocial acceptance is crucial for better coping and medication adherence in the face of HIV and mental illness challenges (Abas et al., 2018; Keene et al., 2022).

Common challenges for individuals with this comorbidity include financial crises, social neglect, sexual abuse, hallucinations and medication adherence issues associated with mental illness. The most significant challenge was financial instability, primarily due to respondents’ low socioeconomic status, limited literacy and difficulties in self-sufficiency. Additionally, many respondents were widowed or divorced, with minimal or no income, which too contributed to the observed financial incapacitation.

To address the challenges and improve coping, many respondents emphasised the importance of financial support, which is particularly significant due to the high poverty levels among this group. HIV itself is known to be a factor contributing to poverty and social well-being, as supported by existing research (Ismail et al., 2016; Rodrigo & Rajapakse, 2010). Implementing financial aid programmes aimed at alleviating poverty among individuals with HIV has been suggested as an intervention to reduce prevalence and enhance their quality of life. This impact has been observed before in a study in Tanzania among adult people with HIV where financial and in-kind incentives were shown to improve outcomes along the HIV care cascade (Czaicki et al., 2017). Furthermore, scaling up public education programmes on the causes, prevention, and risks of HIV and its comorbidities, led by government or religious institutions, can help people avoid acquiring these diseases and assist those already affected in taking better care of themselves. The respondents’ observations point to a need for more comprehensive HIV education programmes, which is particularly relevant considering the rising trend in HIV prevalence in Uganda since 2017 (Uganda-AIDS-Commission, 2017).

### Limitations

The study was carried out in resource-limited areas where some participants usually seek healthcare services irregularly due to socioeconomic factors or geographical location. Meaning there was a likelihood of missing out perspectives of such individuals. To mitigate this (i) we collected data on different clinic days per site and (ii) the healthcare workers blindly called the potential participants to come for review.

Stigma associated with HIV and mental illness could affect some participants to underreport their experiences, particularly in fear of discrimination or negative reactions from others. This could result in an incomplete understanding of the coping strategies and challenges faced by this population. However, we were able to restore participants’ trust by ensuring privacy including the exclusion of their names anywhere in the discussion and on the consent forms.

## Conclusions

This study delved into the coping strategies, challenges and potential interventions for individuals facing the dual burden of HIV and mental illness comorbidity in Southwestern Uganda. The coping strategies employed by these individuals included intuitive actions at the individual level, empathic support from family and community, as well as professional interventions at the healthcare facility level. These strategies provided valuable insights into how patients strive to attain good health and well-being as they live with both HIV and mental illness. However, significant challenges were identified, including medication management issues, vulnerability to abuse (females being abused sexually), social isolation and financial instability, primarily due to poverty. The participants’ valuable input has shed light on potential interventions to address these challenges of poverty, gender inequality and improve the quality of life for those grappling with the comorbidity.

Thus, a more integrated approach to healthcare that addresses both HIV and mental illness simultaneously, rather than managing them independently, is warranted. Particularly, more emphasis should target enhancing psychosocial support, raising awareness about the causes, prevention, and risks of HIV and mental illness comorbidity, alleviating the financial burden on individuals with this comorbidity and community engagement. All these will enhance emotional stability, reduce stigmatisation among patients, and reduce inequalities between individuals and societies. These conclusions conform to the sustainable development goals (1,3,5,10 and 15) adopted by the General Assembly of the United Nations.

## Data Availability

The datasets used and/or analysed during the current study are available from the corresponding author on reasonable request.

## List of abbreviations

PWH: People with HIV
ART: Anti-retroviral therapy
